# The mortality burden related to COVID-19 in 2020 and 2021 - years of life lost and excess mortality in 13 countries and sub-national regions in Southern and Eastern Europe, and Central Asia

**DOI:** 10.3389/fpubh.2024.1378229

**Published:** 2024-06-06

**Authors:** Caoimhe Cawley, Mehtap Çakmak Barsbay, Tolkun Djamangulova, Batmanduul Erdenebat, Šeila Cilović-Lagarija, Vladyslav Fedorchenko, Jonila Gabrani, Natalya Glushkova, Arijana Kalaveshi, Levan Kandelaki, Konstantine Kazanjan, Khorolsuren Lkhagvasuren, Milena Santric Milicevic, Diloram Sadikkhodjayeva, Siniša Skočibušić, Stela Stojisavljevic, Gülcan Tecirli, Natasa Terzic, Alexander Rommel, Annelene Wengler, for the BoCO-19-Study Group

**Affiliations:** ^1^Department of Epidemiology and Health Monitoring, Robert Koch Institute, Berlin, Germany; ^2^Faculty of Health Sciences, Department of Healthcare Management, Ankara University, Ankara, Türkiye; ^3^Public Association “Healthy Future”, Bishkek, Kyrgyzstan; ^4^Department of Health Policy, School of Public Health, Mongolian National University of Medical Sciences, Ulan Bator, Mongolia; ^5^Institute for Public Health of the Federation of Bosnia and Herzegovina, Sarajevo, Bosnia and Herzegovina; ^6^Center for Public Health of the Ministry of Health of Ukraine, Kyiv, Ukraine; ^7^European University of Tirana, Tirana, Albania; ^8^Faculty of Medicine, Al-Farabi Kazakhs National University, Almaty, Kazakhstan; ^9^National Institute of Public Health of Kosova, Pristina, Kosovo; ^10^National Center for Disease Control and Public Health, Tbilisi, Georgia; ^11^Laboratory for Strengthening Capacity and Performance of Health System and Workforce for Health Equity, Institute of Social Medicine, Faculty of Medicine, University of Belgrade, Belgrade, Serbia; ^12^Tashkent Institute of Postgraduate Medical Education, Tashkent, Uzbekistan; ^13^Faculty of Medicine, University of Mostar, Mostar, Bosnia and Herzegovina; ^14^Public Health Institute of the Republic of Srpska, Banja Luka, Bosnia and Herzegovina; ^15^Faculty of Medicine, University of Banja Luka, Banja Luka, Bosnia and Herzegovina; ^16^Ministry of Health, Ankara, Türkiye; ^17^Institute of Public Health of Montenegro, Podgorica, Montenegro

**Keywords:** COVID-19, SARS-CoV-2, burden of disease, mortality, years of life lost

## Abstract

**Introduction:**

Between 2021 and 2023, a project was funded in order to explore the mortality burden (YLL–Years of Life Lost, excess mortality) of COVID-19 in Southern and Eastern Europe, and Central Asia.

**Methods:**

For each national or sub-national region, data on COVID-19 deaths and population data were collected for the period March 2020 to December 2021. Unstandardized and age-standardised YLL rates were calculated according to standard burden of disease methodology. In addition, all-cause mortality data for the period 2015–2019 were collected and used as a baseline to estimate excess mortality in each national or sub-national region in the years 2020 and 2021.

**Results:**

On average, 15–30 years of life were lost per death in the various countries and regions. Generally, YLL rates per 100,000 were higher in countries and regions in Southern and Eastern Europe compared to Central Asia. However, there were differences in how countries and regions defined and counted COVID-19 deaths. In most countries and sub-national regions, YLL rates per 100,000 (both age-standardised and unstandardized) were higher in 2021 compared to 2020, and higher amongst men compared to women. Some countries showed high excess mortality rates, suggesting under-diagnosis or under-reporting of COVID-19 deaths, and/or relatively large numbers of deaths due to indirect effects of the pandemic.

**Conclusion:**

Our results suggest that the COVID-19 mortality burden was greater in many countries and regions in Southern and Eastern Europe compared to Central Asia. However, heterogeneity in the data (differences in the definitions and counting of COVID-19 deaths) may have influenced our results. Understanding possible reasons for the differences was difficult, as many factors are likely to play a role (e.g., differences in the extent of public health and social measures to control the spread of COVID-19, differences in testing strategies and/or vaccination rates). Future cross-country analyses should try to develop structured approaches in an attempt to understand the relative importance of such factors. Furthermore, in order to improve the robustness and comparability of burden of disease indicators, efforts should be made to harmonise case definitions and reporting for COVID-19 deaths across countries.

## 1 Introduction

Burden of disease (BoD) indicators can be used to assess the combined impact of morbidity and mortality on population health by calculating life years lost due to disease and death, enabling the comparison of the impact of different diseases over time and/or across countries (1–4). Global estimates indicate an increase in the burden of non-communicable diseases relative to communicable diseases or injuries over the last 20–30 years in Central and Eastern Europe and Central Asia (5). Before the COVID-19 pandemic, lower respiratory infections were the most notable communicable diseases in these regions, accounting for an estimated 2.2% of the total disability-adjusted life years (DALYs) (2, 5). The availability of population-based research in these regions is limited, with current studies mainly focusing on non-communicable diseases as the primary national prevention priorities (6–8) despite the double communicable and non-communicable burden in certain countries (9). This lack of information renders their health systems vulnerable to the detrimental effects of the COVID-19 or other potential pandemics. Therefore, it is imperative to gather and analyse national data to better inform governments and health organisations of the pandemic’s impact on public health, which will help decision-making processes for mitigating future pandemics.

In response to the national need to evaluate the impact of the pandemic on public health, a consensus COVID-19 disease model was developed and published by the European Burden of Disease Network in 2021 (10). Although numerous countries have already published national estimates of the COVID-19 burden (11–15), it is evident that some regions in Southern and Eastern Europe and Central Asia have yet to provide such estimates, creating a gap in understanding the overall impact of the pandemic on population health. To support this effort, the German Federal Ministry of Health funded a project between 2021 and 2023 under the Global Health Protection Programme. The project aimed to strengthen capacities and data infrastructures for calculating BoD indicators in countries and regions in Southern and Eastern Europe, the Southern Caucasus, and Central Asia, with a particular focus on COVID-19 (16). The resulting project (‘BoCO-19 - The Burden of Disease due to COVID-19. Toward a harmonisation of population health metrics for the surveillance of dynamic outbreaks’) created a scientific network and platform, bringing together public health professionals from 14 different partner institutions. In this paper, we report on work done within the BoCO-19 project to assess the mortality burden due to COVID-19 in 2020 and 2021 by calculating the indicator YLL (Years of Life Lost) for the partner countries and regions, differentiated by age and sex. Furthermore, whilst BoD indicators have not commonly been used in the context of pandemic monitoring, they provide an additional tool that could be used to do so. We, therefore, additionally visualise YLL trends over time in order to explore pandemic patterns in the partner countries and regions.

**Figure 1:**
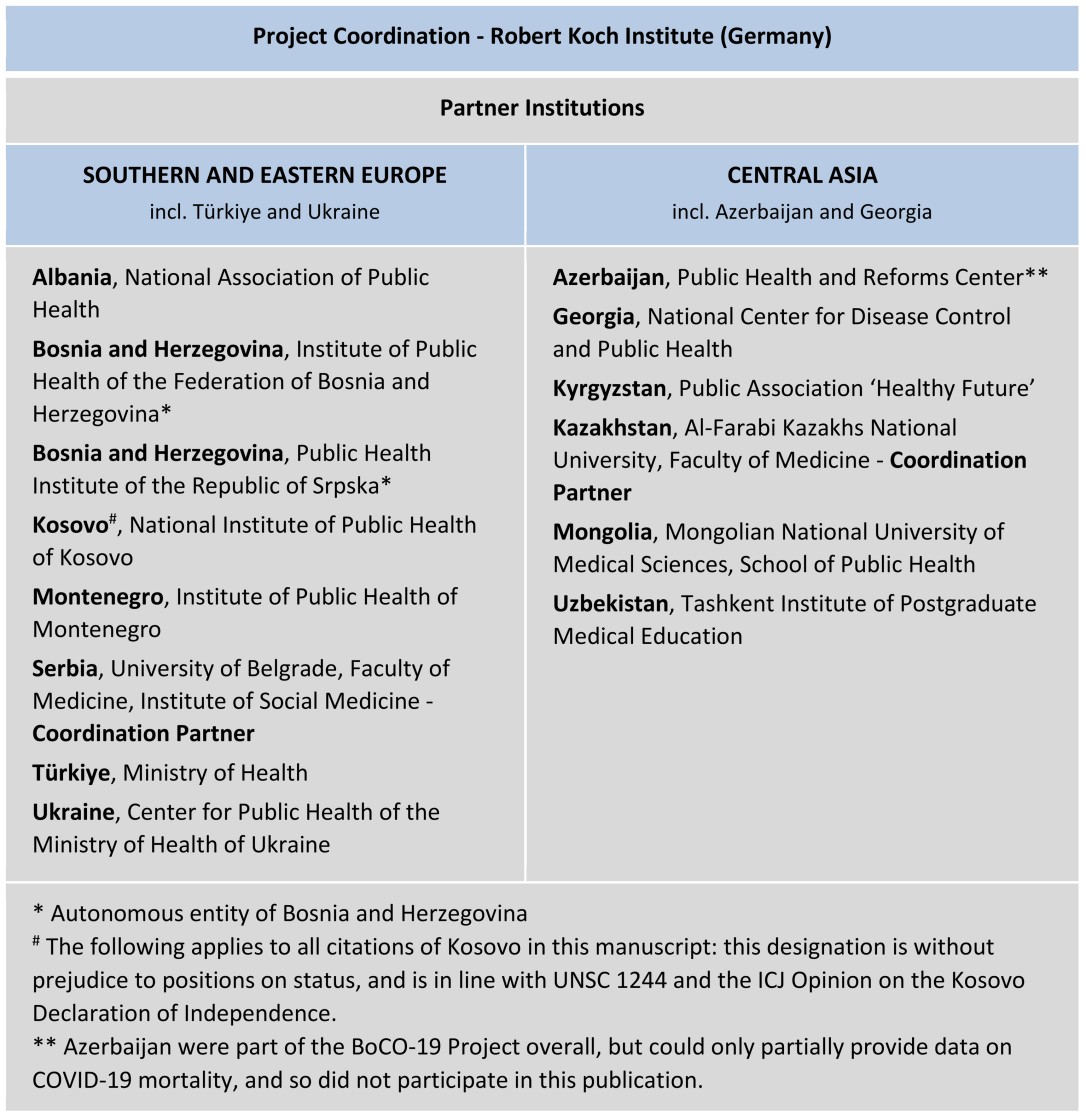
BoCO-19 partner institutions.

The partner institutions that participated in the BoCO-19 project are shown in Figure 1, grouped into two regions (Southern and Eastern Europe and Central Asia). The project was coordinated by the Robert Koch Institute in Berlin, Germany, with regional coordination support from the University of Belgrade, Serbia, and Al-Farabi Kazakhs National University in Almaty, Kazakhstan. Whilst Azerbaijan participated in the project overall, they could provide only partial data on COVID-19 mortality, so results for this country are not included in this publication.

## 2 Materials and methods

### 2.1 Data sources and collection

National data on COVID-19 deaths and population data were collected for the period March 2020 to December 2021 by all partners. All partners reported following the WHO guideline definition for deaths due to COVID-19, which states that a COVID-19 death is one “resulting from a clinically compatible illness, in a probable or confirmed COVID-19 case, unless there is a clear alternative cause of death that cannot be related to COVID disease (e.g., trauma)” (17). However, the implementation of this guideline varied in practise. For example, some countries reported that a COVID-19 death was only counted if ICD-10 code ‘U07.1’ (COVID-19 confirmed by laboratory testing) had been reported, whereas in others code ‘U07.2’ (COVID-19 diagnosed clinically or epidemiologically, without laboratory test confirmation) was also counted (see Table 1). Furthermore, in some countries and sub-national regions deaths not only ‘due to’ COVID-19 (i.e., COVID-19 was the main, primary cause of death), but also deaths ‘with’ COVID-19 were counted.

**Table 1:** Data sources, data availability and definitions of COVID-19 deaths by countries and sub-national regions.

Country or sub-national region	Data source for COVID-19 deaths^1^	Data source all-cause mortality numbers	Data source population numbers	ICD-10-codes used in practice for COVID-19 deaths^1^	Deaths counted in reporting statistics (‘due to’ or ‘with’ COVID-19, or both)
Albania	Ministry of Health and Social Protection	Institute of Statistics, INSTAT	Institute of Statistics, INSTAT	U07.1/U07.2	Due to
Federation of Bosnia & Herzegovina	Federal Office of Statistics (Federation of Bosnia and Herzegovina)	Federal Office of Statistics (Federation of Bosnia and Herzegovina)	Federal Office of Statistics (Federation of Bosnia and Herzegovina)	U07.1/U07.2	Due to
Republic of Srpska	Public Health Institute of the Republic of Srpska	Republic of Srpska Institute of Statistics	Republic of Srpska Institute of Statistics	U07.1/U07.2	Both
Kosovo	National Institute of Public Health of Kosovo	The Kosovo Agency of Statistics (KAS)	Civil Registration Agency of Kosovo	U07.1/U07.2	Both
Montenegro	Institute of Public Health of Montenegro (Monitoring System of Infectious disease)	Statistical Office of Montenegro	Statistical Office of Montenegro	U07.1	Both
Serbia	Statistical Office of the Republic of Serbia	Statistical Office of the Republic of Serbia	Statistical Office of the Republic of Serbia	U07.1/U07.2	Due to
Türkiye	Ministry of Health of Türkiye	National Statistical Office	National Statistical Office	U06.0/U07.3	Both
Ukraine	Public Health Center of the Ministry of Health of Ukraine	State Statistics Service of Ukraine	UN Department of Economic and Social Affairs, Population Division	U07.1/U07.2	Unknown
Georgia	Geostat/NCDC Georgia	National Statistics Office (Geostat)	National Statistics Office (Geostat)	U07.1	Both
Kazakhstan	Ministry of Health	Office for National Statistics	Office for National Statistics	U07.1/U07.2	Unknown
Kyrgyzstan	National Statistic Committee	National Statistic Committee	National Statistic Committee	U07.1/U07.2	Both
Mongolia	Health Development Center, Ministry of Health	Health Development Center, Ministry of Health	Health Development Center, Ministry of Health	U07.1	Both
Uzbekistan	Ministry of Health	National Statistics Committee, Ministry of Health	National Statistics Committee, Ministry of Health	U07.1	Due to

^1^All partners reported following the WHO guideline on coding and counting COVID-19 deaths (17).

To facilitate data collection by all partners in a uniform manner, data collection templates were created (templates available upon request). The data on COVID-19 deaths were collected by sex, five-year age-group (uppermost age-group 85 years and older), and month. In Albania, Türkiye, and Kazakhstan, death data were not available in five-year age-groups so different age-groupings were used (see Supplementary Table 1). In Uzbekistan, 2021 data were disaggregated by sex and age-group only, not by month. Furthermore, in Albania, whilst overall COVID-19 death data were disaggregated by sex and ten-year age-group, daily data were not. Therefore, monthly numbers were generated by aggregating daily figures, and an assumption was made that the monthly distribution by sex and age-group was the same as for the whole period from March 2020 to December 2021.

### 2.2 Data analysis

YLL were calculated according to standard BoD methodology, using the Global Burden of Disease (GBD) 2019 residual life expectancy (RLE) at the time of death [see 11, 18, 19 for more details, (20)]. The GBD 2019 table provides average RLE estimates in five-year age-groups (except for young children aged less than 1 year and 1–4 years old, for whom separate life expectancies are provided), which matched the age-groups used for BoCO-19 data collection, and hence could be applied in our calculations. For the youngest age-group (0–4 years), we used the GBD RLE estimate for those aged less than 1 year, whilst for the oldest age-group (≥85) we assigned the average RLE of those aged 85–90 years. For Albania, Türkiye, and Kazakhstan, where different age-groups were used, we chose the estimate for the youngest five-year ‘sub age-group’ within broader age-groups as the reference life expectancy.

For each country or region, we calculated the absolute number of YLL by sex, age-group, and month, as well as the unstandardized rates per 100,000 population, and age-standardised rates using the World Standard Population. Because the choice of standard population can influence the results obtained (21), we also calculated age-standardised rates using the European Standard Population, as a sensitivity analysis (results available upon request).

### 2.3 Excess mortality

Excess mortality summarises the surplus (or deficit) in all-cause (rather than cause-specific) deaths in any given year, compared to what might have been expected based on an analysis of numbers of all-cause deaths in preceding years (the so-called ‘baseline’). Excess mortality therefore provides a more comprehensive measure of the total impact of the pandemic on population health, capturing COVID-19 deaths that were not correctly diagnosed and reported, as well as deaths from other causes that may have occurred due to indirect effects of the pandemic (e.g., deaths from chronic diseases due to lack of access to treatment and care during the pandemic). Different methodological decisions have to be made when calculating excess mortality (e.g., which baseline period is observed, and how trends and seasonal variations are incorporated) (22).

Within BoCO-19, due to differences between countries in the level of granularity of all-cause mortality data, a simple approach was taken. All partners estimated the number of excess deaths in their country or sub-national region in 2020 and 2021, using all-cause mortality data from 2015 to 2019 as a baseline. For both years (i.e., both 2020 and 2021) the same baseline period was chosen so that the expected number of deaths was not influenced by pandemic processes in the countries. The calculations were done in Excel using exponential smoothing, taking trend and seasonality into account.

### 2.4 Ethical statement

At the Robert Koch Institute as a government agency, the legal department assumes the role of an Institutional Review Board in the ethical review of non-interventional studies that do not themselves collect medical data on individuals. In BoCO-19 only aggregated data is used that has been collected by governmental agencies as part of mandatory reporting on notifiable diseases. Hence, BoCO-19 uses existing information on COVID-19 cases and deaths in the population that is disaggregated only by age, sex, and time prior to the data provision and analysis. Such data on infectious diseases are used to describe and assess the epidemiological situation and are forwarded to international bodies such as the WHO in order to analyse population health on an international level. No additional individual data on humans is collected and interventions are neither feasible nor planned. Hence, the data used in BoCO-19 are official aggregated case counts and not individual medical information. The legal department of the Robert Koch Institute has therefore stated that an ethical vote is not required for the data usage in question (Reference No. 1.09.04/0011#0259).

## 3 Results

### 3.1 Mortality burden

The numbers of YLL lost per death in each country or sub-national region and year ranged between 15 and 30 YLL per death (see Table 2). Generally, in both 2020 and 2021, more COVID-19 deaths were reported in countries and regions in Southern and Eastern Europe compared to Central Asia, resulting in higher YLL rates per 100,000 (both unstandardized and age-standardised –Table 2). Sensitivity analyses showed that the use of the European Standard Population rather than the World Standard Population for the calculation of age-standardised YLL resulted in higher rates across all countries and regions (due to the older age-structure of the European Standard Population), but the patterns between countries and regions remained similar.

**Table 2:** Years of life lost related to reported COVID-19 deaths by countries and sub-national regions in 2020 and 2021 (total YLL, unstandardised and age-standardised YLL rates, YLL per death).

	Total YLL (absolute number)		YLL per 100,000 (unstandardised rate)		YLL per 100,000 (age-standardised rate)		YLL per death	
	2020	2021	2020	2021	2020	2021	2020	2021
Southern and Eastern Europe (including Türkiye and Ukraine)								
Albania	34,571	59,600	1,222	2,133	731	1,260	29	29
Fed. Bosnia and Herzegovina	59,420	127,085	2,720	5,860	1,399	2,901	22	22
Kosovo	30,180	38,903	1,602	2,065	1,259	1,650	19	24
Montenegro	15,994	37,703	2,574	6,089	1,430	3,262	23	22
Serbia	229,860	580,980	3,332	8,501	1,549	3,741	22	21
Republic of Srpska	38,350	78,012	3,375	6,914	1,480	2,912	22	21
Türkiye	579,058	1,659,432	693	1,985	544	1,548	26	25
Ukraine	214,692	1,779,568	489	4,053	265	2,160	25	24
Central Asia (including Georgia)								
Georgia	41,299	216,255	1,109	5,809	898	3,275	15	22
Kazakhstan	99,506	396,702	534	2,101	1,491	2,089	29	29
Kyrgyzstan	53,934	48,810	820	723	773	863	30	27
Mongolia	0	51,514	0	1,555	0	1,905	0	26
Uzbekistan	18,429	21,105	53	61	61	77	30	25

Source: see Table 1, own calculations.

In nearly all countries, the YLL burden was greater in 2021 compared to 2020, though the extent of the increase varied (e.g., in Ukraine: unstandardized YLL per 100,000 population was approximately 8 times higher in 2021 compared to 2020. In Kosovo: this increase was approximately 1.3 times higher – see Table 2). In Kyrgyzstan and Uzbekistan, the YLL burden was similar in both years. Mongolia was the only country with no reported COVID-19 deaths in 2020.

**Figure 2:**
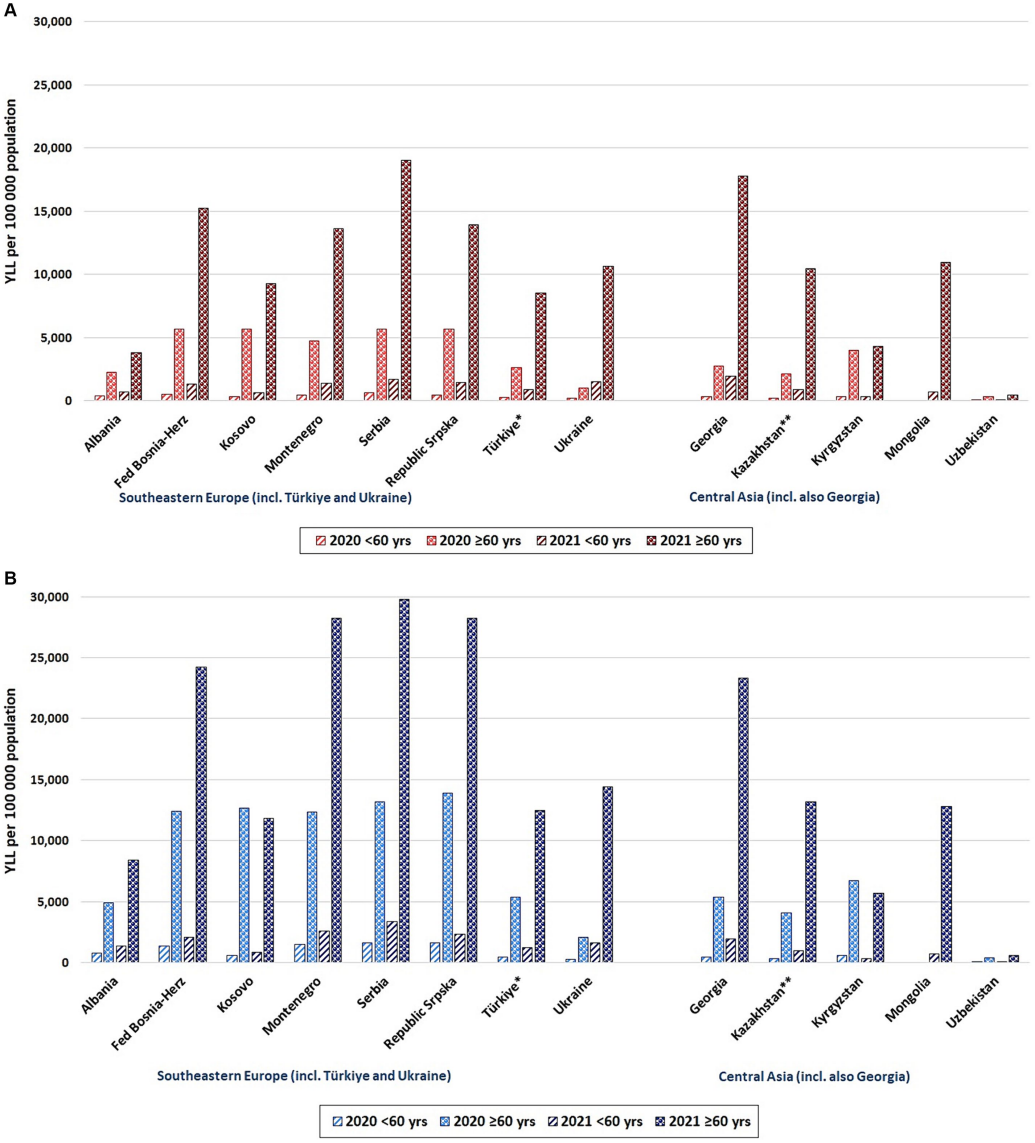
Years of life lost related to reported COVID-19 deaths: rates per 100,000 population by year (2020, 2021) and age-group (<60 years, ≥60 years) by countries and sub-national regions. **(A)** Females **(B)** Males. ^*^Türkiye: age-groups are <65, ≥65. ^**^Kazakhstan: age-groups are <61, ≥61.

In nearly all countries and regions, in both years and across age-groups, YLL rates per 100,000 were higher amongst men than amongst women (Figure 2) (For detailed country-specific charts on YLL rates by sex and five-year age-groups, see Supplementary Figure 1). For example, in Montenegro in 2021, amongst those aged ≥60 years, the YLL rate was 2.1 times higher amongst men compared to women; for those aged <60 years, the rate was 1.9 times higher amongst men compared to women. In Central Asian countries, however, the difference in rates between men and women was less stark compared to most countries in Southern and Eastern Europe. For example, in Kazakhstan in 2021, amongst those aged ≥60 years, the YLL rate was 1.3 times higher amongst men than women; for those aged <60 years the rate was only 1.1 times higher amongst men than women.

Figure 2 clearly demonstrates that in both years and all countries and regions, YLL rates per 100,000 were higher amongst older individuals aged ≥60 compared to those aged <60. However, the degree to which the burden was greater amongst older individuals varied. For example, in Albania in 2021, the YLL rate was 5.8 times greater amongst females ≥60 compared to females <60 (amongst males: 6.3 times greater) whilst in Mongolia the differences in YLL rates were considerably higher (16.6 times amongst females vs. 18.0 times greater amongst males, when comparing those aged ≥60 to those aged <60).

**Figure 3:**
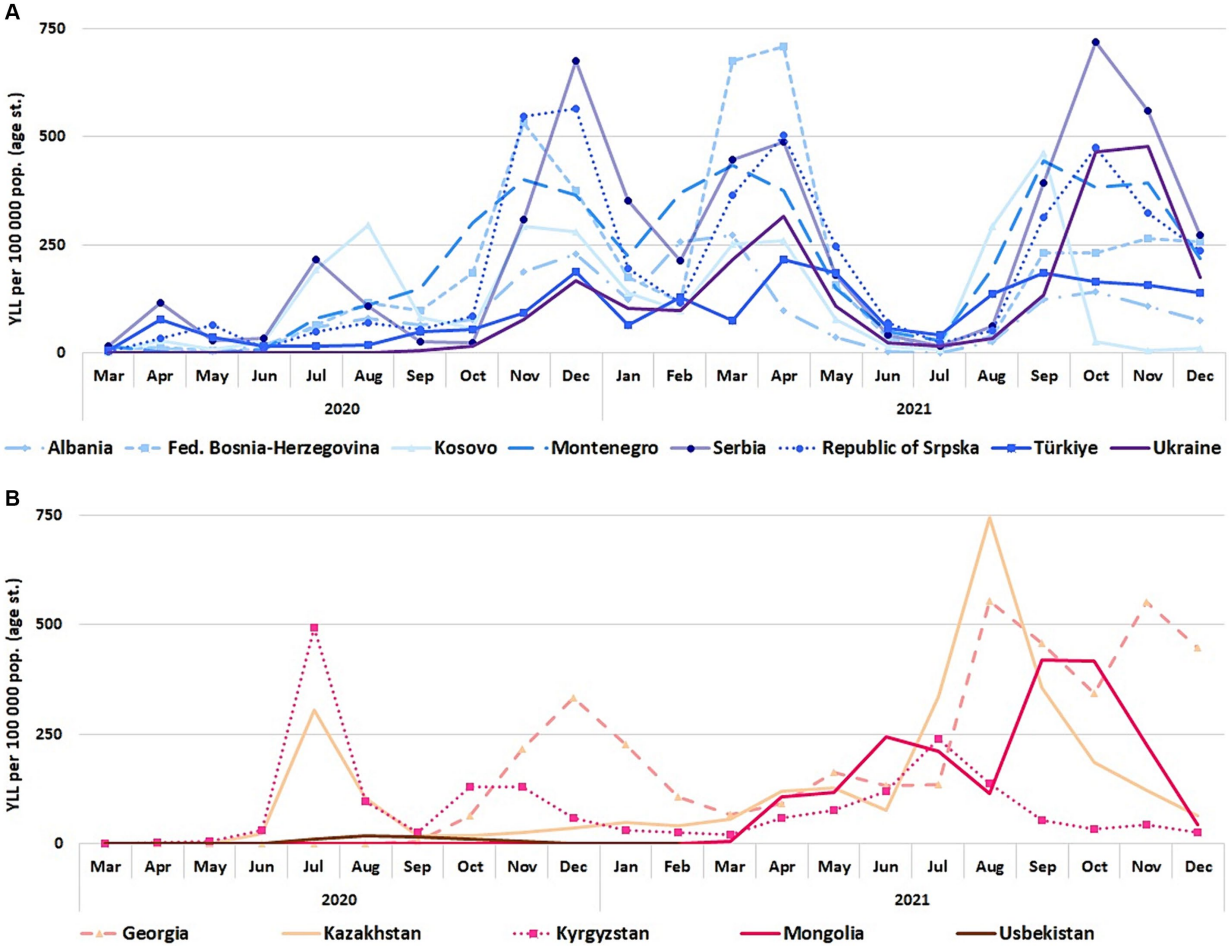
COVID-19 YLL age-standardized rates per 100,000 population (World Standard Population) by month, March 2020 to December 2021. **(A)** BoCO-19 countries in Southern and Eastern Europe (including Türkiye and Ukraine) **(B)** BoCO-19 countries in Central Asia (including Georgia).

The analysis of COVID-19 YLL rates over time (Figure 3) showed that especially in Southern and Eastern Europe, peak rates were experienced in the autumn and winter months of 2020 and 2021, and in the spring of 2021. This was not the case in Central Asia, where YLL rates started to increase later than in most Southern and Eastern European countries: in Central Asia YLL peaks were less pronounced in 2020 (exception: peaks in Kyrgyzstan and Kazakhstan in July 2020, and Georgia in December 2020). Overall in Central Asia and in Georgia, the main share of YLL fell into the summer and autumn months of 2021 (note: for Uzbekistan, there are no 2021 data disaggregated by month). During pandemic peaks or waves, the distribution of the YLL burden by age and sex reflected the distribution seen for the analysis period overall, as seen in Figure 2 (i.e., the YLL burden was greatest amongst men and amongst older individuals).

### 3.2 Excess mortality analyses

**Figure 4:**
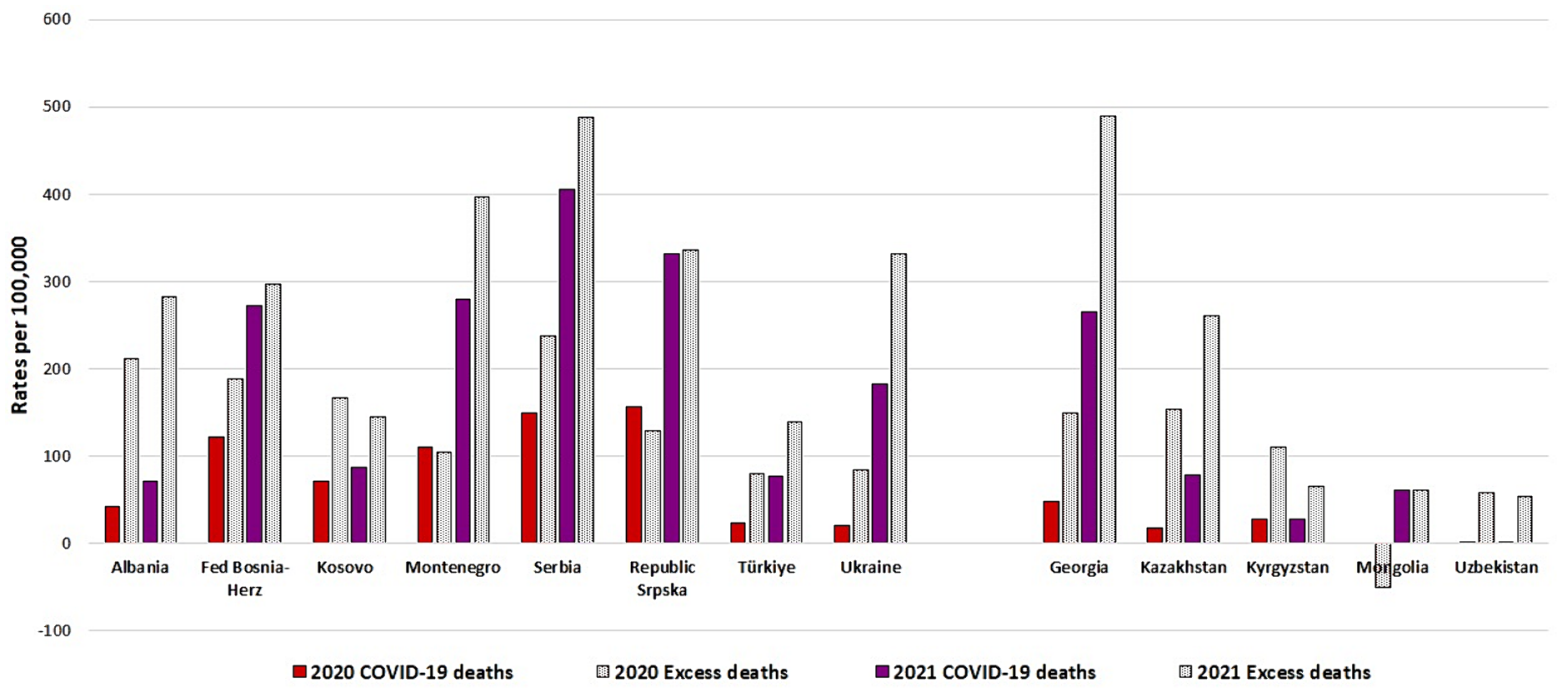
Number of COVID-19 deaths per 100,000, number of excess deaths per 100,000 by country/region and year (2020, 2021), as calculated within the BoCO-19 Project.

Figure 4 displays for 2020 and 2021, the number of COVID-19 deaths per 100,000 population in the observed countries and regions, as well as the crude rates of excess or deficit deaths, as calculated within the project. Differences between the rates of COVID-19 deaths and the rates of excess deaths differed by year and by country/region. In many of the BoCO-19 countries (e.g., in the Central Asian countries), in both years excess death rates were considerably higher than reported COVID-19 death rates. In Mongolia, as the only exception, in 2020 fewer deaths than expected were reported, whilst in 2021 the rates of reported COVID-19 deaths and excess deaths were similar (see Figure 4). In some countries, for example in the Federation of Bosnia-Herzegovina and the Republic of Srpska, differences in rates of reported COVID-19 deaths and excess deaths were less marked. Data on the numbers of COVID-19 deaths, all-cause deaths and excess death estimations are provided in Supplementary Table 2.

## 4 Discussion

In this paper, we investigated the reported mortality burden related to COVID-19, calculating and presenting for the first time COVID-19 YLL estimates for 14 different countries or sub-national regions, and examining data on excess mortality. Broadly speaking, we found that Southern and Eastern Europe officially reported higher COVID-19 mortality, resulting in higher unstandardised and age-standardised YLL rates per 100,000, when compared to most countries in Central Asia. When comparing the reported COVID-19 YLL burden in the BoCO-19 countries and regions with other countries in Western Europe (which generally suffered a high COVID-19 burden), the picture is varied. For example, in France in 2020, 1,472 COVID-19 DALY were reported per 100,000 population, with YLL accounting for 99% of that burden (23). The corresponding figure for the Netherlands in 2020 was 1,640 DALY per 100,000 population (with YLL again accounting for more than 99% of the burden) (24). In the BoCO-19 region, countries with a lower reported COVID-19 mortality burden in 2020 compared to France and the Netherlands included Albania, Georgia, Kazakhstan, Kyrgyzstan, Türkiye, Ukraine, and Uzbekistan, whilst the other BoCO-19 countries had a larger reported burden.

Disentangling reasons for differences in the reported COVID-19 mortality burden between countries is difficult because many factors play a role. A fundamental issue to consider is how COVID-19 deaths are defined and counted. Within the BoCO-19 countries and sub-national regions, most countries reported counting both of the main COVID-19 ICD-10 codes as a case (U07.1: COVID-19 confirmed by laboratory testing, U07.2: COVID-19 diagnosed clinically or epidemiologically, without laboratory test confirmation). However, some countries, such as Montenegro, Georgia, and Uzbekistan, only count laboratory-confirmed cases (code U07.1). There are additional nuances in how deaths are counted, because in some countries, only deaths where the primary underlying cause of death is COVID-19 are counted (deaths ‘due to’ COVID-19), whilst in other countries, all deaths amongst COVID-19 cases are counted (death either ‘due to’ or ‘with’ COVID-19), and in some countries it is unclear what is included in the statistics (see Table 1). In Uzbekistan, only those with laboratory-confirmed infection are counted as COVID-19 cases, and only those dying due to COVID-19 are counted as deaths, which are some possible reasons for the relatively lower reported COVID-19 mortality burden in Uzbekistan over the observed period. Such issues highlight the importance of adhering to standardized guidelines on the coding and counting of deaths, such as those published by the WHO (17), in order to ensure consistency and comparability across countries. Furthermore, where national mortality data are available in a timely manner and by mutually exclusive causes of death, this aids in the identification or differentiation between deaths which were ‘due to’ or ‘with’ COVID-19 (10). Thus ensuring high levels of quality and consistency in data collection and reporting mechanisms across countries is of great importance.

Within our analyses, differences in the extent of COVID-19 testing strategies between countries, as well as differences in completeness of death reporting may also account for some of the varying patterns seen. According to the SCORE Assessment Summaries tool published by the WHO, which summarises the strengths and weaknesses of in-country health information systems (25), levels of completeness of death registration vary between some of the BoCO-19 countries and sub-national regions (reports available for Albania, Mongolia, Türkiye, and Ukraine) (26). For example, using data from 2013 to 2018, the completeness of death registration is estimated to be 90% for Mongolia and Ukraine, and 98% for Albania and Türkiye. However, our calculated results on excess mortality show that in many BoCO-19 countries and regions, there were large differences between reported COVID-19 deaths per 100,000 population and excess death estimations, suggesting possible under-diagnosis or under-reporting of COVID-19 deaths during the pandemic, and/or relatively large numbers of deaths due to indirect effects on other causes of death. When compared with estimates published by the WHO (27), our excess mortality estimates for 2020 are largely similar. For 2021 however, the WHO estimates for many of the BoCO-19 countries and regions are even larger than our estimates, lending further support to the idea that there was under-reporting or under-counting of COVID-19 deaths over the pandemic period (see Supplementary Table 2).

Other broader factors which could explain the different COVID-19 mortality burdens between the BoCO-19 countries and sub-national regions include differences in the extent and implementation of public health and social measures to control the spread of COVID-19 infection, differences in vaccination rates, and/or differences in the underlying age-structures and health status of the population (e.g., with regard to comorbidities) (28–30). For example, in many Western Balkan countries, public health measures to control the spread of SARS-CoV-2 were very effective during the first half of 2020. During this period, many countries there suffered a lower COVID-19 burden compared to Western European countries. However, measures were less strict later in 2020 and in 2021, when the burden in the Western Balkan countries increased (31). Another contributing factor may have been that vaccination rates (one dose of vaccine or more) were lower in many Western Balkan countries compared to some Western European countries such as Germany, Italy, and France (31, 32).

In nearly all BoCO-19 countries and sub-national regions, with the exception of Kyrgyzstan and Uzbekistan, the reported mortality burden of COVID-19 was considerably greater in 2021 compared to 2020. In Mongolia in 2020, strict public health and social measures were implemented to prevent the spread of COVID-19, including bans on public gatherings, and major restrictions on internal and international travel and movement (30, 33), and neither COVID-19 deaths nor excess mortality were reported in Mongolia in 2020. In contrast, in 2021 the first COVID-19 deaths were reported and the respective YLL burden rose to 1,555 per 100,000 population. In Ukraine, there was also a very large increase in the reported YLL burden between 2020 and 2021. This is likely due to a number of factors, including higher testing rates in 2021, but also the appearance of the Delta strain in summer 2021, which was more severe than previously circulating variants (34, 35). The Delta variant was primarily responsible for the COVID-19 wave in Ukraine from late summer of 2021 until the end of that year (36). In other countries and regions (e.g., Albania, Kosovo), increases in YLL rates between 2020 and 2021 were not as marked.

The finding that the reported COVID-19 burden in the BoCO-19 countries and sub-national regions was greater amongst men and older individuals mirrors patterns reported in many other countries globally (11, 12, 37, 38). In Mongolia, there were particularly stark differences in COVID-19 YLL rates between those aged less than 60 and those aged older than 60. The finding that the reported COVID-19 mortality burden was generally higher in countries in Southern and Eastern Europe compared to Central Asia may be related to population age structures, because many Central Asian countries have a younger population compared to European countries (39), and correspondingly a smaller proportion of the population at higher risk for severe COVID-19.

Within the analyses conducted, we were able to plot trends in YLL rates by month over the period of observation (March 2020–December 2021), which shows that YLL can be used alongside other traditional indicators, such as mortality rates, to provide a view of trends over time. One aspect of the YLL indicator is that it gives greater weight to deaths at younger ages (i.e., more YLL accrued by deaths at younger ages). Accordingly, analyses could be stratified to compare the proportion of deaths with the proportion of YLL by age-group, which may show that despite smaller numbers of deaths in younger age-groups, the relative burden when viewed in terms of YLL may be substantial.

### 4.1 Limitations

Important limitations of our analyses relate to the heterogeneity of the data, particularly with regard to the coding and counting of COVID-19 deaths in the different BoCO-19 countries and sub-national regions. Within the time-frame of this project, we were unable to undertake analyses that considered the differences in these definitions in a more detailed way, as well as other important differences between countries and regions (e.g., differences in healthcare infrastructures and the implementation of public health measures, differences in population age structures, etc.), which might help to explain some of the results seen. Future cross-country analyses should take a structured approach to try and assess and analyse the relative importance of some of these factors. Some strengths of our analyses relate to the inclusion of many different countries and regions, and the use of a harmonised approach in our calculations. Despite some of the limitations in the data, the results present a first view of the COVID-19 mortality impact in the different regions and have shed light upon aspects of the data and analyses which could be improved upon in the future.

## 5 Conclusion

The analyses presented here provide a broad overview of the mortality burden of COVID-19 in 2020 and 2021 in 13 different countries and sub-national regions in Southern and Eastern Europe, and Central Asia. Our results suggest that overall, the mortality burden was greater in Southern and Eastern Europe, particularly in the West Balkan countries. However, heterogeneity in the data and other country-specific differences may have influenced our results, and it was not possible within the timeframe of the project to specify the most likely reasons for differences. To improve the robustness and comparability of the burden of disease indicators in the future, efforts should be made to (i) ensure the timely availability of high-quality data, including disaggregated by key parameters such as by age, sex, and time (for example by using electronic data transfer systems), (ii) better harmonise definitions for cases and deaths across countries, and (iii) ensure that the reporting of cases and deaths is as complete as possible.

## Author’s note

This designation is without prejudice to positions on status, and is in line with UNSCR 1244 and the ICJ Opinion on the Kosovo Declaration of Independence.

## Data availability statement

The data analyzed in this study is subject to the following licences/restrictions: data from 14 partners, restrictions differ. Requests to access these datasets should be directed to wenglera@rki.de.

## Ethics statement

Ethical approval was not required for the study involving humans in accordance with the local legislation and institutional requirements. Written informed consent to participate in this study was not required from the participants or the participants' legal guardians/next of kin in accordance with the national legislation and the institutional requirements.

## Author contributions

CC: Methodology, Project administration, Supervision, Visualization, Writing – original draft, Writing – review & editing. MB: Formal analysis, Writing – review & editing. TD: Formal analysis, Writing – review & editing. BE: Formal analysis, Writing – review & editing. ŠC-L: Formal analysis, Writing – review & editing. VF: Writing – review & editing. JG: Formal analysis, Writing – review & editing. NG: Formal analysis, Writing – review & editing. AK: Writing – review & editing. LK: Formal analysis, Writing – review & editing. KK: Formal analysis, Writing – review & editing. KL: Formal analysis, Writing – review & editing. MSM: Formal analysis, Writing – review & editing. DS: Writing – review & editing, Formal analysis. SSk: Writing – review & editing. SSt: Formal analysis, Writing – review & editing. GT: Formal analysis, Writing – review & editing. NT: Formal analysis, Writing – review & editing. AR: Formal analysis, Methodology, Supervision, Writing – review & editing, Funding acquisition, Project administration. AW: Formal analysis, Writing – review & editing, Methodology, Supervision, Visualization, Writing – original draft.

## BoCO-19 Study Group members

Rakhat Aidaraliev, Aline Anton, Timur Aripov, Vesna Bjegovic Mikanovic, Mehtap Çakmak Barsbay, Caoimhe Cawley, Seila Cilovic Lagarija, Kairat Davletov, Tolkun Djamangulova, Batmanduul Erdenebat, Vladyslav Fedorchenko, Jonila Gabrani, Azhar Giniyat, Natalya Glushkova, Pranvera Kaçaniku-Gunga, Levan Kandelaki, Konstantine Kazanjan, Maia Kereselidze, Atadjan Khamrayev, Natalia Kopaleishvili, Oksana Koshalko, Nailya Kozhekenova, Besfort Kryeziu, Khorolsuren Lkhagvasuren, Kledia Nakuci, Olena Nesterova, Dariia Oharova, Alexander Rommel, Natasa Rosic, Diloram Sadikkhodjayeva, Milena Santric Milicevic, Yuliya Semenova, Nabil Seyidov, Sinisa Skcibusic, Milica Stanisic, Aleksandar Stevanović, Stela Stojisavljevic, Gulnaz Tanatarova, Gulcan Tecirli, Natasa Terzic, Elena von der Lippe, Annelene Wengler, Oleksandr Zeziulin.
