# Supplementary material for: The mortality burden related to COVID-19 in 2020 and 2021 - years of life lost and excess mortality in 13 countries and sub-national regions in Southern and Eastern Europe, and Central Asia
Source: Front Public Health. 2024 Jun 6;12:1378229. doi: 10.3389/fpubh.2024.1378229 (PMC11187286; doi:10.3389/fpubh.2024.1378229)
Supplement: Supplementary file 1 [file Data_Sheet_1.docx]

*Supplementary Material*

# Tables

**Supplementary Table 1**: Age-groups for calculating COVID-19 YLL in the BoCO-19 project

| General (five-year) | Albania | Türkiye | Kazakhstan |
| --- | --- | --- | --- |
| 0-4 | 0-9 | 0-14 | <1 |
|  |  |  | 1-14 |
| 5-9 |  |  |  |
| 10-14 | 10-19 |  |  |
| 15-19 |  | 15-24 | 15-17 |
|  |  |  | 18-30 |
| 20-24 | 20-29 |  |  |
| 25-29 |  | 25-34 |  |
| 30-34 | 30-39 |  |  |
|  |  |  | 31-40 |
| 35-39 |  | 35-44 |  |
| 40-44 | 40-49 |  |  |
|  |  |  | 41-50 |
| 45-49 |  | 45-54 |  |
| 50-54 | 50-59 |  |  |
|  |  |  | 51-60 |
| 55-59 |  | 55-64 |  |
| 60-64 | 60-69 |  |  |
|  |  |  | 61-70 |
| 65-69 |  | 65-74 |  |
| 70-74 | 70 and older |  |  |
|  |  |  | 71-80 |
| 75-79 |  | 75-84 |  |
| 80-84 |  |  |  |
|  |  |  | 81 and older |
| 85 and older |  | 85 and older |  |

**Supplementary Table 2:** COVID-19 deaths, excess deaths, and population numbers for 2020 & 2021

|  |  | COVID-19 deaths | | All cause deaths | | Excess deaths - own estimations | | Excess deaths WHO estimations* | | Population | |
| --- | --- | --- | --- | --- | --- | --- | --- | --- | --- | --- | --- |
|  |  | **2020** | **2021** | **2020** | **2021** | **2020** | **2021** | **2020** | **2021** | **2020** | **2021** |
| Southern and Eastern Europe | **Albania** | 1,181 | 2,036 | 27,605 | 30,507 | 5,993 | 8,011 | 6,016 | 15,102 | 2,829,741 | 2,829,741 |
|  | **Fed. Bonsnia Herzegovina*** | 2,665 | 5,895 | 26,026 | 29,086 | 4,115 | 6,438 | - | - | 2,184,680 | 2,168,602 |
|  | **Kosovo*** | 1,339 | 1,655 | 12,987 | 13,019 | 3,146 | 2,743 | - | - | 1,883,804 | 1,883,804 |
|  | **Montenegro** | 682 | 1,729 | 7,293 | 9,152 | 653 | 2,456 | 737 | 3,524 | 621,306 | 619,211 |
|  | **Serbia** | 10,352 | 27,740 | 116,850 | 135,901 | 16,395 | 33,342 | 16,724 | 53,166 | 6,899,126 | 6,834,326 |
|  | **Republic Srpska*** | 1,783 | 3,741 | 16,582 | 19,002 | 1,465 | 3,794 | - | - | 1,136,274 | 1,128,309 |
|  | **Türkiye** | 19,170 | 65,198 | 507,938 | 565,594 | 66,802 | 118,139 | 76,726 | 210,154 | 83,614,362 | 84,680,273 |
|  | **Ukraine** | 8,754 | 75,129 | 616,769 | 714,263 | 36,729 | 136,144 | 43,126 | 187,836 | 43,909,667 | 40,997,698 |
| Central Asia | **Georgia** | 1,807 | 9,860 | 50,537 | 59,906 | 5,549 | 18,146 | 5,738 | 22,994 | 3,722,716 | 3,708,610 |
|  | **Kazakhstan** | 3,379 | 14,836 | 162,613 | 183,357 |  |  | 31,218 | 83,269 | 18,650,590 | 18,879,552 |
|  | **Kyrgyzstan** | 1,814 | 1,834 | 39,972 |  | 7,279 | 4,386 | 7,421 | 14,348 | 6,580,166 | 6,692,064 |
|  | **Mongolia** | 0 | 2,014 | 15,922 | 19,931 | -1,685 | 2,014 | -1,804 | 796 | 3,312,275 | 3,312,275 |
|  | **Uzbekistan** | 614 | 861 | 175,625 | 174,541 | *no age stratified data* | | 15,794 | 30,620 | 34,558,891 | 34,558,891 |

Data sources: Table 1 (main paper), WHO: https://www.who.int/data/sets/global-excess-deaths-associated-with-covid-19-modelled-estimates

*WHO estimates are available for all BoCO-19 countries and regions except for the Federation of Bosnia and Herzegovina, Republic of Srpska, and Kosovo. The WHO results were generally in line with our own estimations, though in some cases the WHO numbers were considerably higher (e.g., higher excess mortality in 2021: almost double in Albania and Türkiye, three times as high in Kyrgyzstan). This may be due to different modelling approaches for all-cause as well as excess mortality. As national or sub-national data on all-cause mortality was available to all partners, eliminating the need to use modelled estimates, the partners jointly decided to use our own excess deaths estimates for the purposes of the paper.

# Figures

**Figure 1**: COVID-19 YLL rates per 100,000 population for BoCO-19 countries, by sex, and age-groups (2020+2021 combined). Note: scales on x-axis differ.

a) Southern and Eastern Europe

b) Central Asia (incl. Georgia)
